# Supplementary material for: Physical Activity, Mental Health and Wellbeing of Adults within and during the Easing of COVID-19 Restrictions, in the United Kingdom and New Zealand
Source: Int J Environ Res Public Health. 2022 Feb 4;19(3):1792. doi: 10.3390/ijerph19031792 (PMC8835233; doi:10.3390/ijerph19031792)
Supplement: Supplementary file 1 [file ijerph-19-01792-s001.zip › ijerph-1576920-supplementary.pdf]

Supplementary Table S1: Changes in exercise behaviours (positive, negative, no change) from T1 to T2 by sex, age groups, ethnicity, long-term health condition and country.

	Negative change (n=552)	Relative risk compared to no change	No change (n=2330)	Positive change (n=480)	Relative risk compared to no change
**Sex**					
Male	120 (12.9%)	REF	686 (73.5%)	127 (13.6%)	REF
Female	428 (17.7%)	1.36 (1.09, 1.71)*	1637 (67.8%)	349 (14.5%)	1.18 (0.93, 1.48)
**Age group**					
18-29 years	90 (22.6%)	REF	240 (60.2%)	69 (17.3%)	REF
30-39 years	113 (19.3%)	0.84 (0.61, 1.17)	368 (62.7%)	106 (18.1%)	0.96 (0.68, 1.37)
40-49 years	115 (16.3%)	0.65 (0.47, 0.90)*	490 (69.3%)	102 (14.4%)	0.77 (0.54, 1.09)
50-59 years	136 (18.1%)	0.70 (0.51, 0.96)*	533 (70.8%)	84 (11.2%)	0.54 (0.38, 0.79)*
60-69 years	68 (11.0%)	0.41 (0.29, 0.60)*	469 (75.5%)	84 (13.5%)	0.64 (0.44, 0.93)*
70-79 years	29 (10.8%)	0.39 (0.25, 0.63)*	209 (78.0%)	30 (11.2%)	0.53 (0.33, 0.85)*
80+ years	1 (4.2%)	0.16 (0.02, 1.20)	19 (79.2%)	4 (16.7%)	0.89 (0.28, 2.76)
**Ethnicity**					
White	514 (16.2%)	REF	2218 (69.8%)	448 (14.1%)	REF
Polynesian	15 (24.2%)	1.52 (0.82, 2.82)	42 (67.7%)	5 (8.1%)	0.84 (0.33, 2.16)
Indian	2 (25.0%)	0.88 (0.33, 2.37)	22 (59.5%)	10 (27.0%)	2.17 (1.01, 4.67)*
Asian	4 (13.8%)	0.80 (0.27, 2.39)	20 (69.0%)	5 (17.2%)	1.63 (0.60, 4.43)
Black	2 (25.0%)	1.71 (0.31, 9.49)	4 (50.0%)	2 (25.0%)	1.69 (0.31, 9.32)

Mixed	6 (28.6%)	1.68 (0.61, 4.60)	12 (57.1%)	3 (14.3%)	0.96 (0.26, 3.48)
Other	6 (24.0%)	1.97 (0.72, 5.38)	12 (48.0%)	7 (28.0%)	3.26 (1.25, 8.47)*
**Long-term health condition**					
Yes	164 (20.2%)	REF	523 (64.3%)	127 (15.6%)	REF
No	379 (15.4%)	0.68 (0.55, 0.84)*	1748 (70.9%)	338 (13.7%)	0.80 (0.63, 1.01)
**Country**					
United Kingdom	215 (16.1%)	REF	865 (65.0%)	251 (18.9%)	REF
New Zealand	337 (16.6%)	0.91 (0.75, 1.11)	1,464 (72.1%)	229 (11.3%)	0.51 (0.42, 0.63)*

*Significantly difference between items (p < 0.001).

Note: REF, Reference value; T1, Timepoint 1; T2, Timepoint 2
